# Who Benefits from Family Psychoeducation for Relatives of Adults with Major Depressive Disorder? Findings from a Randomized Controlled Trial

**DOI:** 10.3390/jcm15114118

**Published:** 2026-05-26

**Authors:** Ida Schou Ipsen, Claudio Csillag, Stephen Fitzgerald Austin, Maj Vinberg

**Affiliations:** 1 The Early Multimodular Prevention and Intervention Research Institution (EMPIRI), Psychiatric Center North Zealand, Copenhagen University Hospital—North Zealand, 3400 Hillerød, Denmark; ida.schou.ipsen@regionh.dk (I.S.I.); claudio.csillag@regionh.dk (C.C.); 2 Department of Clinical Medicine, Faculty of Health and Medical Sciences, University of Copenhagen, 2200 Copenhagen, Denmark; 3 Mental Health Services, Region Zealand East, 4000 Roskilde, Denmark; stephen.fitzgerald.austin@regionh.dk; 4 Department of Psychology, University of Copenhagen, 1353 Copenhagen, Denmark

**Keywords:** major depressive disorder, family psychoeducation, caregivers, family functioning

## Abstract

**Background**: Major depressive disorder (MDD) affects not only patients but also their relatives, who often carry substantial emotional and practical responsibilities. Family psychoeducation has shown benefits in several psychiatric conditions, yet its effects on relatives of adults with MDD remain insufficiently documented. **Aim**: We aimed to examine whether a brief group-based family psychoeducation program improves relatives’ well-being and perceived family functioning compared with an active social-support control condition and to explore whether intervention response varies across caregiver subgroups. **Methods**: Relatives of patients with MDD were enrolled in a two-center randomized controlled trial and allocated to either a four-week psychoeducation program or a structurally matched social-support group. Outcomes were assessed at baseline, post-intervention, and 9-month follow-up using the WHO-5 Well-Being Index (WHO-5), the Family Attitude Scale (FAS), and the Family Assessment Device (FAD). Repeated-measures ANCOVA models tested time × group interactions, with and without adjustment for age and gender. **Results**: Eighty-nine relatives were included (*n* = 43 intervention; *n* = 46 control). No significant intervention effects were observed on well-being (WHO-5) or family attitudes (FAS). A significant time × group interaction was found only for the FAD affective involvement subscale, with short-term improvement in the intervention group compared with deterioration in the control group. Subgroup analyses suggested a heterogeneous pattern of response, with more consistent patterns of improvement among older relatives (≥50 years), non-partner relatives, and those with a history of psychiatric treatment, while effects appeared more limited among partners and younger participants. Women showed worsening communication in the intervention group, whereas men demonstrated improvements in selected well-being and general functioning outcomes. **Conclusions**: The intervention showed limited effects at the whole-sample level, but exploratory subgroup analyses suggested that responsiveness to brief family psychoeducation may vary according to caregiver characteristics. These findings support further investigation of more targeted psychoeducational approaches for relatives of adults with MDD.

## 1. Introduction

Globally, the one-year prevalence of major depressive disorder (MDD) has been estimated at 3.4%, highlighting the considerable burden associated with the condition [1]. In Scandinavia, specialist-care registry data show similar one-year prevalence rates, with 3.2% of the Danish population, 3.1% of the Swedish population, and 4.4% of the Norwegian sample receiving a diagnosis of MDD [1]. Cumulative incidence increases across adulthood, reaching 6.7% in Denmark, 5.4% in Sweden, and 9.0% in Norway [1]. The Scandinavian populations seem to resemble other Western populations in key demographic and clinical characteristics, suggesting that these regional figures reflect patterns relevant to comparable high-income settings [1].

Mental illness not only impacts the individual level but also affects the whole family, including the relatives who take on daily caregiving responsibilities. In a large national caregiver study of informal caregivers of individuals with mental illness, 72.1% reported a high caregiving burden, indicating that extensive demands are common in families affected by mental illness [2]. This burden was closely linked to characteristics of the ill family member, including symptom stability, medication adherence, and insight, which caregivers had to manage in everyday life. These demands tend to accumulate, particularly when relatives serve as the primary source of support, which may shape how they experience their role and organize daily family routines [2].

Beyond these practical responsibilities, MDD may also affect broader family functioning and relational dynamics. Qualitative studies suggest that relatives often struggle to interpret and respond to fluctuating depressive symptoms while continuously adapting to changes in mood, behavior, and everyday functioning in the affected family member [3,4]. Relatives frequently report emotional strain, uncertainty, and disruptions to established family roles and routines, which may affect communication, boundaries, and daily interactions within the family [3,4].

Given these challenges, psychosocial interventions targeting relatives have increasingly received attention across psychiatric disorders. A recent review found that psychosocial interventions, especially psychoeducation, often improve the relatives’ burden and well-being, though evidence certainty is low to moderate, and interventions vary widely [5]. While not specific to MDD, this literature targets relatives’ needs and highlights that effects are unlikely to be uniform across families.

Family psychoeducation is well established in schizophrenia treatment, with research showing it reduces relapse, rehospitalization, and improves family functioning and caregiver burden [6]. For MDD, the evidence base is smaller, although a recent review suggested potential benefits for depressive symptoms, relapse prevention, and family-related outcomes [7]. While most psychoeducational trials for MDD have focused on patient outcomes, only a small number have assessed effects on relatives themselves. However, findings regarding outcomes among relatives themselves remain limited and inconsistent. One study of multifamily psychoeducation for chronic depression found no significant change in relatives’ psychological distress but did show short-term improvements in family functioning and relatives’ depressive symptoms [8].

Family psychoeducation offers a structured framework to assist relatives in navigating the practical and emotional challenges associated with mental illness. Core elements typically include clarifying the nature of the disorder and working with families to improve communication, coping strategies, and problem-solving skills. The aim is to support relatives in managing illness-related demands without positioning the family as the object of treatment. However, given the heterogeneity of caregiving roles and family contexts, such interventions may not be expected to benefit all relatives in the same way. Therefore, the present trial investigates whether a brief group-based family psychoeducation program can improve relatives’ well-being and perceived family functioning compared with an active social-support control condition. In addition to examining effects at the whole-sample level, the study explores whether responsiveness to the intervention varies according to caregiver characteristics.

## 2. Methods

### 2.1. Study Design

This study is part of a double-center randomized controlled trial (Mental Health Centre North Zealand and Mental Health Centre Amager), originally designed to evaluate outcomes in patients with MDD and their adult relatives [9]. The present study reports outcomes from the relatives. The trial employed a parallel-group design in which relatives were randomized to either a structured family psychoeducation program (intervention) or a social-support group (control). Investigators and raters conducting assessments were blinded to group allocation.

All relatives completed assessments at three time points. Baseline data were collected between April 2015 and February 2017. Post-intervention assessments were conducted immediately after completion of the four-week intervention, and 9-month follow-up assessments were obtained approximately nine months later.

### 2.2. Participants

A total of 89 relatives were enrolled in the study, with 43 allocated to the intervention group and 46 to the control group. Relatives were recruited in connection with the inclusion of patients diagnosed with MDD at two outpatient centers affiliated with the University of Copenhagen (Mental Health Centre North Zealand and Mental Health Centre Amager), as well as from private-practice psychiatrists in the North Zealand region [9]. Patients identified a relative whom they considered emotionally important and willing to participate in the study.

Patients met ICD-10 diagnostic criteria for MDD, verified by a psychiatrist using the MINI International Neuropsychiatric Interview. Eligible patients were aged 18–75 years and were required to be in remission or partial remission at the time of inclusion, defined as a score below 13 on the Hamilton Depression Rating Scale (HAM-D17) [9].

Eligible relatives were adults aged 18–75 years with sufficient proficiency in Danish. Relatives were defined as individuals living with, or in regular close contact with, the patient and whom the patient identified as emotionally important. This definition ensured inclusion of partners, family members, or other significant persons involved in the patient’s everyday life [9].

### 2.3. Randomization and Blinding

After baseline assessments, relatives were individually randomized to the intervention or control group using computer-generated allocation. Randomization procedures ensured that assessors remained blinded throughout the study. Relatives were instructed not to disclose their allocation during interviews to maintain blinding integrity.

### 2.4. Interventions

- Intervention group: Family psychoeducation
  Relatives in the intervention group attended a four-week structured psychoeducation program delivered in small groups of up to six participants. The patients with MDD did not participate in the sessions. Each weekly meeting lasted approximately 120 min and consisted of a brief lecture followed by a longer interactive component. The lecture introduced core topics such as depression, family emotional dynamics, and expressed emotion. The interactive part focused on practicing problem-solving strategies for managing high-emotion situations within the family. Discussions were supported by fictional cases designed to help participants apply the material to their own everyday challenges.
  Sessions were facilitated by psychiatric nurses or psychologists experienced in delivering manualized psychoeducation for mood disorders. Two group leaders conducted each psychoeducation group. The program drew partly on the McFarlane multifamily model [10] and on existing research on reducing expressed emotion, with particular attention to criticism as a key element in families affected by unipolar depression.
- Control group: Social-support meetings
  Relatives assigned to the control condition attended four group meetings matching the intervention group in duration and overall structure but without psychoeducational content. The meetings provided a moderated forum for sharing experiences with other families. The group leader acted as facilitator rather than instructor and did not present any structured material, although participants could request suggestions for discussion topics. Each group was led by one trained clinician. After each session, the facilitator recorded the themes discussed.

### 2.5. Quality Assurance

All sessions in both arms were videotaped. For the intervention group, an independent assessor reviewed recordings to ensure adherence to key elements of the treatment manual.

### 2.6. Outcome Measures

Since the present analysis focuses solely on relatives, all outcomes are based on self-report instruments completed by the relatives themselves. The primary measures were the WHO-5 Well-Being Index (WHO-5), the Family Attitude Scale (FAS), and the Family Assessment Device (FAD). The WHO-5 assesses psychological well-being, with higher scores indicating better well-being. The FAS measures criticism and hostility within the family, and the FAD captures several domains of family functioning, including communication, problem solving, affective responsiveness, affective involvement, and general functioning, where higher scores indicate poorer functioning or a more negative family climate. Sociodemographic data were collected at baseline.

### 2.7. Assessment Schedule

Relatives completed all outcome measures at baseline, immediately after the intervention period, and at 9-month follow-up. All assessments were conducted by trained raters blinded to group allocation. Raters participated in regular consensus meetings to maintain inter-rater reliability.

### 2.8. Statistical Analysis

Repeated-measures ANCOVA models were used to examine time × group interactions across the three assessment points. Analyses were conducted both unadjusted and adjusted for the covariates age and gender. All tests were two-sided. Subgroup analyses were conducted as exploratory and hypothesis-generating analyses. Age-stratified analyses used a cut-off at 50 years to reflect broad life-course differences between earlier and later adulthood, including differences in caregiving context and competing work- and family-related responsibilities. Given the limited sample size, the study was not powered to detect subgroup differences, and these analyses should therefore be interpreted cautiously. No formal correction for multiple comparisons was applied, as the subgroup analyses were intended to explore potential patterns of differential responsiveness for future investigation.

### 2.9. Ethical Considerations

The study was conducted in accordance with the principles of the Helsinki Declaration and the CONSORT guidelines. All participants received written and oral information about the study and provided written informed consent prior to participation. Participants were informed that they could withdraw at any time without consequences for ongoing care. No adverse events related to study participation were expected. The trial is registered with the Danish Data Protection Agency and on ClinicalTrials.gov under the ID number NCT02348827, registered 5 January 2015.

## 3. Results

### 3.1. Study Characteristics

A total of 89 relatives were randomized, with 46 allocated to the control group and 43 to the intervention group. Baseline data were available for 88 participants (control *n* = 46; intervention *n* = 42). Follow-up data were obtained from 71 participants at post-intervention (control *n* = 34; intervention *n* = 37) and from 51 participants at the 9-month follow-up (control *n* = 27; intervention *n* = 24). The groups were comparable in terms of age, gender distribution, educational attainment, employment status, psychiatric history, and relationship to the patient (Table 1).

### 3.2. Effects on Primary Outcomes

**Figure 1:**
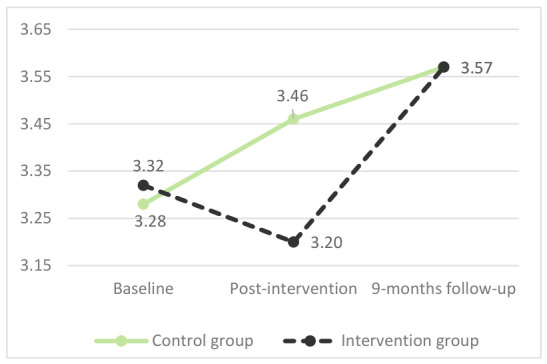
Changes in affective involvement across time by intervention group (data from Table 1). Affective involvement was measured using the Family Assessment Device (FAD). Values represent mean scores at baseline, post-intervention, and 9-month follow-up.

Descriptive outcome measures across time points are presented in Table 1, while results from repeated-measures ANCOVA are shown in Table 2. Changes in affective involvement are illustrated in Figure 1.

Repeated-measures ANCOVA revealed no significant time × group interactions at the whole-sample level for the WHO-5 total score across the main contrasts (baseline to post-intervention, post-intervention to 9 months, and baseline to 9 months). Thus, no consistent intervention-specific effects were observed—all *p* values > 0.05 (Table 2). The overall time × group interaction effect for the WHO-5 total score was negligible in magnitude (partial η^2^ = 0.003).

For the Family Attitude Scale, no significant time × group interactions were observed (Table 2). The corresponding interaction effect was small in magnitude (partial η^2^ = 0.033).

On the Family Assessment Device (FAD; range 1–4, with higher scores indicating poorer family functioning), significant time × group interactions were observed only on the affective involvement subscale. From baseline to post-intervention (Model 1: *p* = 0.022; Model 2: *p* = 0.041), scores in the intervention group showed a slight improvement, whereas the control group demonstrated a deterioration (Figure 1). The observed interaction effect corresponded to a moderate effect size (partial η^2^ = 0.111). No significant interactions were detected on the remaining FAD subscales—communication, problem solving, affective responsiveness, or general functioning—across any of the tested time intervals (Table 2).

Taken together, no intervention-specific effects were observed for the WHO-5 or FAS, and on the FAD, the affective involvement scale showed a significant group-specific effect.

### 3.3. Subgroup Analyses

#### 3.3.1. Gender

Among women (control *n* = 14, intervention *n* = 18), significant time × group interactions were observed for the FAD communication subscale (range 1–4; higher scores indicate poorer communication) from baseline to the 9-month follow-up (Model 1: *p* = 0.009; Model 2: *p* = 0.010) and across all three time points (Model 1: *p* = 0.033; Model 2: *p* = 0.035). Scores in the control group improved from 2.58 at baseline to 2.41 at 9-month follow-up (*n* = 14, 8), whereas the intervention group showed a deterioration from 2.39 to 2.67 (*n* = 18, 6).

**Figure 2:**
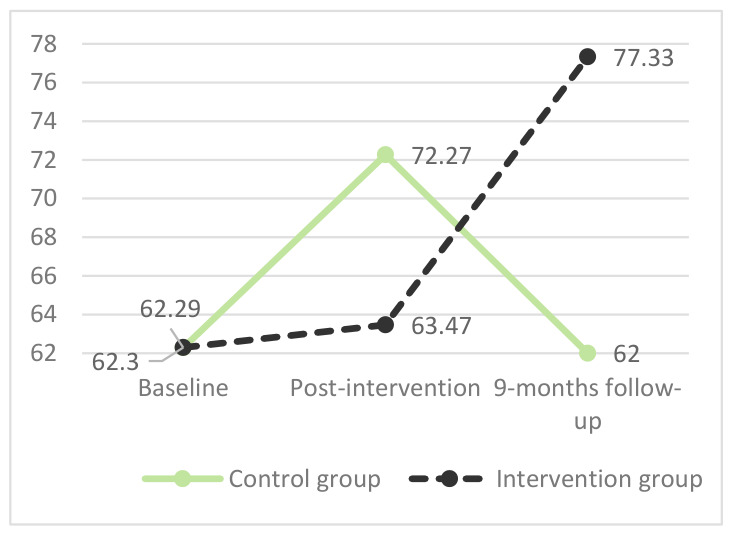
Changes in well-being over time among men by intervention group. Well-being was measured using the World Health Organization 5-item Well-Being Index (WHO-5). Values represent mean scores at baseline, post-intervention, and 9-month follow-up.

Among men (control *n* = 21, intervention *n* = 18), repeated-measures analyses indicated intervention-specific effects on selected outcomes. For the WHO-5 total score (range 0–100; higher scores indicating greater well-being), a significant/borderline time × group interaction was observed from baseline to post-intervention (Model 1: *p* = 0.031; Model 2: *p* = 0.061), reflecting greater improvement in the control group compared with the intervention group (Figure 2). Thus, findings among men were not uniform across outcomes, as the primary WHO-5 contrast favored the control condition, whereas other exploratory outcomes suggested intervention-related improvements at longer follow-up intervals.

**Figure 3:**
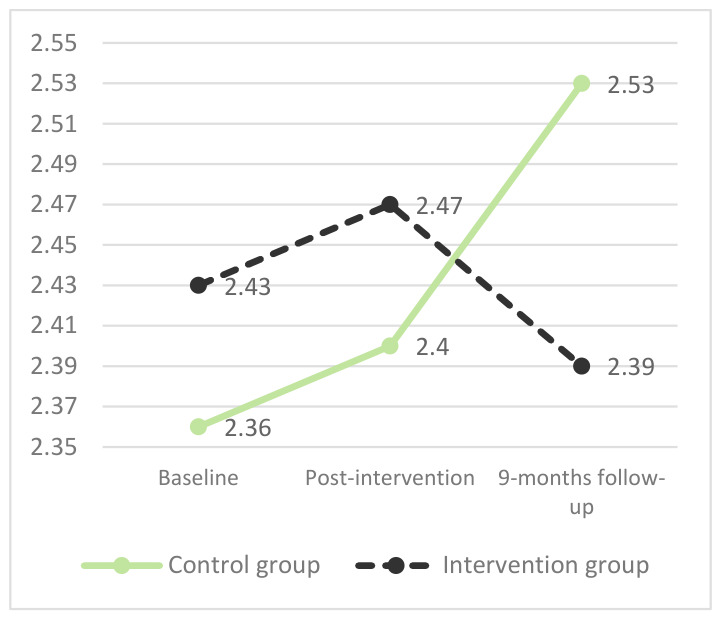
Changes in general functioning over time among men by intervention group. General functioning was measured using the Family Assessment Device (FAD). Values represent mean scores at baseline, post-intervention, and 9-month follow-up.

On the FAD general functioning subscale (range 1–4; higher scores indicate poorer general functioning), significant interactions were found from baseline to 9-months follow-up (Model 1: *p* = 0.018; Model 2: *p* = 0.024) and across all three time points (Model 1: *p* = 0.045; Model 2: *p* = 0.051), reflecting improvement in the intervention group and worsening in the control group (Figure 3).

#### 3.3.2. Age

**Figure 4:**
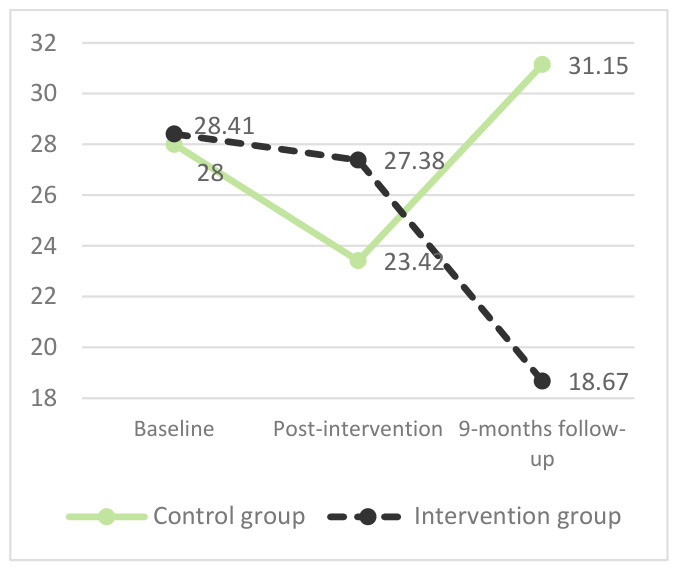
Changes in family attitudes over time among participants younger than 50 years by intervention group. Family attitudes were measured using the Family Attitude Scale (FAS). Values represent mean scores at baseline, post-intervention, and 9-month follow-up.

In participants younger than 50 years (control *n* = 28, intervention *n* = 18), intervention-specific differences were limited to the Family Attitude Scale (FAS). Non-significant trends were observed from baseline to 9-month follow-up (Model 1: *p* = 0.095; Model 2: *p* = 0.053) and across all three time points (Model 1: *p* = 0.143; Model 2: *p* = 0.086), reflecting improvements in the intervention group compared with deterioration in controls (Figure 4), with lower scores indicating less criticism and hostility within the family.

Among participants aged 50 years and older (control *n* = 15, intervention *n* = 25), repeated-measures analyses indicated several intervention-specific effects.

For the WHO-5 total score (range 0–100; higher scores indicate better well-being), a time × group interaction from baseline to the 9-month follow-up (Model 1: *p* = 0.045; Model 2: *p* = 0.082) showed larger improvements in the intervention group. Scores increased from 67.68 at baseline to 77.11 at follow-up (*n* = 25, 18), compared with a smaller rise in controls from 66.67 to 68.33 (*n* = 15, 12).

On the FAD affective involvement subscale (range 1–4; higher scores indicate less healthy emotional involvement), borderline interactions from baseline to post-intervention (Model 1: *p* = 0.076; Model 2: *p* = 0.081) were observed. Scores increased in the control group from 3.30 to 3.56 (*n* = 10, 9), while the intervention group showed a slight improvement, moving from 3.51 to 3.45 (*n* = 15, 12).

By contrast, one outcome favored the control condition. On the FAD communication subscale (range 1–4, with higher scores indicating poorer communication), a significant baseline-to-9-month interaction was observed (Model 2: *p* = 0.008), with scores improving in the control group from 2.63 to 2.40, whereas scores in the intervention group worsened from 2.44 to 2.60 over follow-up.

#### 3.3.3. Relationship to the Patient

For relatives who were spouses or partners (control *n* = 28, intervention *n* = 20), no consistent intervention-specific effects were observed across the primary outcomes. Among non-partner relatives (children, parents, siblings, grandparents, and others) (control *n* = 18, intervention *n* = 23), exploratory analyses suggested selective differences across some measures of well-being and family functioning.

For the Family Attitude Scale (FAS; range 0–100; higher scores indicate greater perceived criticism), a baseline-to-post-intervention interaction was observed (Model 1: *p* = 0.031; Model 2: *p* = 0.081). Scores decreased from 21.43 to 16.66 in the intervention group (*n* = 23, 20), although the reduction was larger in the control group from 22.83 to 13.36 (*n* = 18, 14).

On the FAD communication subscale (range 1–4; higher scores indicate poorer communication), a baseline-to-9-month interaction was observed (Model 2: *p* = 0.045). Scores in the intervention group increased from 2.43 to 2.63 (*n* = 15, 6), while controls remained relatively stable from 2.49 to 2.45 (*n* = 11, 6).

Finally, on the FAD affective involvement subscale (range 1–4; higher scores indicate less healthy emotional involvement), significant interactions from baseline to post-intervention (Model 1: *p* = 0.003; Model 2: *p* = 0.032) showed that both groups worsened, though the deterioration was smaller in the intervention group. Scores increased from 3.41 to 3.55 (*n* = 15, 6) in the intervention group, compared with a larger rise from 3.24 to 3.60 in controls (*n* = 13, 6).

#### 3.3.4. Previous Psychiatric Treatment

Relatives with a history of psychiatric treatment showed more intervention-related differences across outcomes compared with those without such a history. Among relatives without previous treatment, significant interactions were limited to the FAD problem-solving subscale from baseline to post-intervention (Model 1: *p* = 0.038; Model 2: *p* = 0.081). On this scale (range 1–4; higher scores indicate poorer problem-solving), controls improved from 1.84 to 1.74 (*n* = 21, 18), whereas scores in the intervention group worsened from 1.68 to 1.89 (*n* = 18, 15).

By contrast, in relatives with a history of psychiatric treatment, several interactions were observed. For WHO-5 total score (range 0–100; higher scores indicate better well-being), interactions across all three time points (Model 1: *p* = 0.033; Model 2: *p* = 0.026) reflected greater improvements in the intervention group, which increased from 54.33 at baseline to 66.80 post-intervention and 68.67 at 9-month follow-up (*n* = 12, 10, 6), compared with more modest improvements in controls from 52.50 to 58.00 and 64.00 (*n* = 8, 6, 5). At the item level, the overall time × group differences appeared to be driven primarily by WHO-5 item 1 (feeling cheerful and in good spirits) and item 4 (rested on waking), both showing improvements in the intervention group compared with controls.

### 3.4. Summary

At the whole-sample level, the intervention did not produce consistent effects on well-being or on indicators of family climate, and only a single domain of family functioning—affective involvement—showed a short-term benefit. However, the subgroup analyses revealed a heterogeneous pattern of responses, suggesting differential responsiveness to the intervention. While effects were not uniform within subgroups, certain patterns emerged. Older relatives (≥50 years), non-partner relatives, and relatives with a history of psychiatric treatment showed intervention-related improvements across selected well-being and family-functioning outcomes across selected outcomes, although findings were not consistent across all measures. Women showed unfavorable changes in family communication, whereas men demonstrated improvements in selected well-being and within-family functioning measures. Overall, the findings indicate that the intervention’s effects were limited at the aggregate level but more pronounced and variable within specific caregiver subgroups. Taken together, the subgroup findings point toward potentially differential responsiveness across caregiver characteristics rather than uniformly distributed intervention effects. However, these patterns should be interpreted cautiously, given the exploratory nature of the analyses and the limited sample size.

## 4. Discussion

This randomized controlled trial examined the effects of a brief group-based family psychoeducation program for relatives of adults with MDD, with a particular focus on relatives’ own well-being and perceived family functioning. At the whole-sample level, limited between-group differences were observed, with no consistent improvements observed in well-being or family climate compared with the active social-support control condition. The only overall intervention-specific effect was a short-term improvement in affective involvement. However, exploratory subgroup analyses suggested a heterogeneous pattern of response across caregiver subgroups, indicating that patterns of response appeared to vary according to caregiver characteristics such as gender, age, relationship to the patient, and previous psychiatric treatment. While these subgroup findings should be interpreted cautiously due to the limited sample size and exploratory analyses, they may point toward clinically relevant variation in responsiveness across caregiving contexts.

### 4.1. Comparison with Other Studies

The intervention did not produce significant effects on global well-being (WHO-5) or perceived criticism and hostility within the family (FAS) at the whole-sample level. However, the subgroup analyses suggest that this aggregate picture may conceal variation in response patterns across caregiver groups. Taken together, the findings indicate that brief psychoeducational interventions for relatives of adults with MDD may have limited broad effects when evaluated across heterogeneous samples, while some subgroup analyses identified intervention-related improvements in specific caregiver groups.

A systematic review by Brady et al., 2016 [11] found limited and mixed evidence that family psychoeducation benefits relatives of people with major depressive disorder. Of the ten studies, only six looked at relatives’ well-being or burden, and only lower-quality studies showed clear positive effects. More robust studies had mixed or no significant results. The review also noted that intervention timing and baseline burden may influence outcomes. When relatives’ initial distress was low, psychoeducation during non-acute illness phases had little effect on overall well-being. With respect to family attitudes and expressed emotion, the review found no consistent evidence that depression-focused psychoeducational interventions lead to improvements in these domains [11]. This provides important context for the present null findings on the Family Attitude Scale and suggests that changes in perceived criticism and hostility may not represent the primary mechanism through which brief psychoeducation affects relatives in MDD [11].

Baseline burden and intervention timing may be moderators in the present study, as the patients had to be in (partial) remission at inclusion. Consequently, relatives may have experienced lower caregiving burden than relatives of patients in more acute depressive phases, potentially limiting the scope for measurable intervention-related improvement. This may have reduced the scope for improvement in relatives’ outcomes. Baseline well-being and family functioning were generally high and may therefore explain the largely null findings. Mean WHO-5 total scores at baseline were relatively high in both groups (control: 63.57; intervention: 64.93), approaching levels typically reported in population samples (approximately 68–70) [12]. Family Attitude Scale scores were low in both groups (means ≈25 on the 0–120 scale like non-clinical samples (20–30)) [13]. Thus, the relatives reported limited criticism or hostility at study entry, reducing the potential for improvement and possibly creating a floor effect for the FAS score.

Similarly, Family Assessment Device results showed families were generally well-functioning in some domains but reported greater difficulties in others. Problem-solving scores were below the clinical cut-off of 2.20 (means 1.85 and 1.77) [14], indicating little room for improvement. However, affective involvement scores were above the cut-off of 2.10 (means 3.28 and 3.32) [14], suggesting more challenges in this area. This pattern may help explain why the intervention-specific effect was observed only for affective involvement, as this was the domain with the greatest potential for improvement.

Against this broader background of mixed findings, the present results are consistent with findings from previous reviews and one of the few randomized controlled trials that have examined outcomes in relatives of adults with MDD [8,11]. In that trial, four sessions of group-based psychoeducation were compared with a single-session nurse-led counselling control, and no significant between-group differences were observed on relatives’ psychological distress, caregiver burden, or perceived criticism.

Overall, the present findings align with the existing evidence in suggesting that the effects of a brief family psychoeducation on relatives of adults with MDD are heterogeneous and context-dependent. Null effects at the whole-sample level should therefore be interpreted cautiously and may mask more selective benefits related to baseline burden, timing, and specific dimensions of family functioning.

Such heterogeneity also appears in the broader caregiver-intervention literature. For severe mental and substance use disorders, psychoeducational interventions have improved carers’ burden and well-being, yet effects vary substantially across outcomes and intervention formats, and average effects are often modest [5]. Although these findings may not directly apply to depression, they support the interpretation that brief family psychoeducation may affect specific subgroups of relatives differently rather than uniformly across samples. In this light, the present study’s main contribution may be less the absence of a general effect and more the indication that responsiveness may differ across caregiving contexts and caregiver characteristics.

### 4.2. Strengths and Limitations

This study has several strengths. First, it is among the few randomized controlled trials to examine the effects of family psychoeducation on relatives of adults with major depressive disorder, with a specific focus on relatives’ own well-being and perceived family functioning. The use of an active control condition matched for group format and contact time strengthens internal validity by reducing the likelihood that observed effects were driven solely by non-specific factors such as social contact or attention. On the other hand, the risk of an improvement in both groups must be considered, as the control group may have benefited from the more non-specific therapeutic ingredients, such as social support, reduced isolation, normalization of experiences, and allowing the participants to share coping strategies. These non-specific therapeutic factors may help explain the limited between-group differences observed in the present study. Accordingly, the intervention did not demonstrate consistent superiority over a structurally comparable social-support condition.

The study benefits from its pragmatic design and recruitment from routine outpatient settings, which enhances ecological validity and relevance to clinical practice. However, the recruitment strategy may also have influenced the findings. Patients were recruited from outpatient settings and were required to be in remission or partial remission at inclusion, which may have resulted in a relatively high-functioning sample with lower baseline caregiving burden and limited room for measurable improvement. Participation additionally required willingness and availability to engage in a group-based intervention, potentially favoring more resourceful and motivated relatives. By including a broad definition of relatives based on emotional closeness rather than formal kinship alone, the study reflects the diversity of family constellations involved in the care of adults with MDD [9]. Further, the outcomes were assessed using well-validated instruments, and blinded outcome assessments were conducted at multiple time points, allowing for examination of both short-term and longer-term effects.

Several limitations should also be considered. Although the sample size was comparable to, or larger than, most previously published trials in this field, the study was not powered to reliably detect small intervention effects or to formally test subgroup differences. Consequently, subgroup findings should be interpreted as exploratory patterns rather than confirmatory. At the same time, several subgroup patterns appeared relatively consistent across conceptually related outcomes, suggesting that the possibility of heterogeneous responsiveness warrants further investigation in larger and adequately powered studies. The limited statistical power increases the risk of type II errors at the whole-sample level and may yield unstable estimates in secondary analyses. Attrition at follow-up assessments was substantial, with data available for only 51 of the 89 randomized participants at the 9-month follow-up. This reduced precision in longer-term estimates and increased the risk of attrition-related bias if participants who completed follow-up differed systematically from those who did not. Reasons for non-completion were not systematically recorded, limiting the ability to determine whether dropout was associated with participant characteristics or intervention responsiveness. Although appropriate statistical methods were applied to account for repeated measurements, missing data remain a potential source of bias. In addition, all outcomes relied on self-report measures, which may be influenced by response shifts, increased awareness, or changes in evaluative standards following psychoeducation [15].

Finally, the intervention was brief and consisted of only four sessions delivered exclusively to relatives without patient participation. Such a format may have limited the intervention’s capacity to influence more stable or deeply embedded interactional patterns within the family system, particularly in domains such as communication and general functioning. More sustained or family-inclusive interventions may therefore be necessary to produce broader or longer-lasting changes in caregiver well-being and family functioning. The heterogeneity of relatives’ relationships to the patient and their baseline levels of burden may also have contributed to variability in intervention responsiveness. These factors should be considered when generalizing the findings and when designing future studies.

### 4.3. Clinical and Research Perspectives

Subgroup analyses revealed a heterogeneous pattern of intervention responses across gender, age, relationship to the patient, and relatives’ psychiatric history. Importantly, these analyses were exploratory, and the study was not powered to detect differential effects across subgroups. Findings should therefore be interpreted with caution and viewed as hypothesis-generating rather than confirmatory. Nevertheless, several subgroup patterns appeared relatively consistent across related outcomes, suggesting that responsiveness to brief family psychoeducation may vary across caregiver groups and warrants further investigation in larger studies.

While counterintuitive, the observed worsening among women on the FAD communication subscale may reflect increased awareness and more critical appraisal of communication difficulties following psychoeducation rather than actual deterioration in family interaction. Interventions that promote reflection on relational processes have previously been associated with heightened evaluative scrutiny and self-criticism, particularly among women, which may influence self-reported outcomes. This interpretation is consistent with longitudinal research indicating that increased reflective engagement can be accompanied by declines in self-rated relational satisfaction without corresponding evidence of objective relational change [16]. Nevertheless, given the exploratory nature of the subgroup analyses, the limited sample size, and the number of comparisons conducted, this finding should be interpreted with caution, as recommended for subgroup analyses in randomized trials [17].

The exploratory analyses suggest that responsiveness to psychoeducation may vary by age group, with several signals of benefit observed among relatives aged 50 years and older, while effects among relatives under 50 were weaker and less uniform. To date, there is no empirical evidence directly examining how age shapes relatives’ responses to psychosocial interventions in the context of severe mental illness, and no studies explicitly address age-related differences in the uptake or effectiveness of such interventions.

However, relevant insights can be drawn from adjacent research on informal caregiving across the life course. A study of adult children providing non-coresidential parental care. Pomeroy & Fiori found that the likelihood of providing informal care increased with age, while the presence of dependent children in the household was associated with a lower probability of caregiving [18]. This life-course pattern aligns with the age stratification applied in the present study, where the threshold of 50 years broadly distinguishes between earlier adulthood, in which employment and parenting responsibilities are more prevalent, and later life stages, where such competing demands are less common. Hence, the weaker and less consistent effects observed among relatives under 50 could potentially reflect greater exposure to life-course-related competing demands rather than differences in motivation or willingness to benefit from the intervention.

In the present study, intervention effects differed by relatives’ relationship to the patient. No consistent intervention-specific effects were observed among spouses and partners, whereas non-partner relatives showed selective improvements in well-being and family functioning. Research on couple-oriented interventions highlights relationship-specific features of partner caregiving that may help to interpret this pattern. A cross-disease review emphasized that partners occupy a uniquely close and interdependent role in illness management, in which psychological well-being, daily functioning, and health-related behaviors were tightly intertwined between patient and partner [19]. It was emphasized that illness-related strain within intimate relationships may affect not only caregiving behaviors but also the relational context in which support is enacted, including communication, reciprocity, and mutual expectations. As a result, psychosocial interventions that do not explicitly engage with these dyadic processes may have limited impact [19]. Hence, the weaker intervention effects observed among partners in the present study may reflect the constraints of intimate caregiving relationships, where support is embedded in ongoing relational dynamics rather than discrete caregiving tasks. In contrast, non-partner relatives may be better positioned to integrate psychoeducational input into their supportive role without the same degree of relational entanglement.

Intervention effects varied according to relatives’ prior psychiatric treatment history. Among relatives without previous psychiatric treatment, intervention-specific effects were minimal and inconsistent, with a single interaction observed for family problem solving that did not favor the intervention. In contrast, relatives with a history of psychiatric treatment showed clearer and more consistent intervention-related improvements, and these were evident across all time points on the WHO-5 total score, mainly driven by increases in positive affect and activation. One possible explanation is that prior psychiatric treatment is associated with greater mental health literacy [20] and may therefore also be more ready to engage with psychoeducational content, facilitating uptake and application of the intervention.

Taken together, the subgroup findings point toward a more targeted understanding of family psychoeducation in MDD. Rather than assuming uniform benefit across relatives, future studies should examine for whom such interventions are most useful and under what circumstances. This may help guide more tailored support strategies based on caregiver life stage, relational position, and prior mental health experience.

## 5. Conclusions

In this randomized controlled trial, a brief family psychoeducation program for relatives of adults with MDD showed limited effects at the whole-sample level, with improvement being primarily confined to affective involvement. However, exploratory subgroup analyses suggested a heterogeneous pattern of responsiveness across caregiver groups. Older relatives (≥50 years), non-partner relatives, and relatives with previous psychiatric treatment showed more consistent signals of improvement across selected outcomes, whereas effects among partners and younger relatives appeared more limited. Although these findings should be interpreted cautiously due to the limited sample size and exploratory subgroup analyses, they point toward the possibility that brief psychoeducation may be more beneficial for some relatives than others. Future studies with larger samples are needed to clarify which caregiver groups are most likely to benefit and whether more targeted psychoeducational approaches may improve support for families affected by MDD.

## Figures and Tables

**Table 1:** Characteristics and health factors of the included relatives to patients with depression according to the intervention group.

	Baseline			Post Intervention			9 Months Follow-Up		
Randomization Group	Control Group (*n* = 46)	Intervention Group (*n* = 42)	*p*-Value	Control Group (*n* = 34)	Intervention Group (*n* = 37)	*p*-Value	Control Group (*n* = 27)	Intervention Group (*n* = 24)	*p*-Value
Sociodemographic:									
Age, mean (s.d.)	45.30 (11.7)	49.2 (13.5)	0.162	*	*	*	*	*	*
Sex, female, *n* (%)	25 (54.30)	25 (58.10)	0.719	*	*	*	*	*	*
Education years, mean (s.d.)	16.36 (3.08)	15.71 (2.67)	0.292	*	*	*	*	*	*
Education level, non-tertiary education, *n* (%)	10 (21.74)	8 (18.60)	0.713	*	*	*	*	*	*
Education level, tertiary education, *n* (%)	36 (78.26)	35 (81.40)	0.713	*	*	*	*	*	*
History of psychiatric treatment, *n* (%)	8 (17.40)	13 (30.23)	0.192	*	*	*	*	*	*
Employed or in education, *n* (%)	40 (86.96)	39 (90.70)	0.203	*	*	*	*	*	*
Not in employment, *n* (%)	6 (13.04)	4 (9.30)	0.203	*	*	*	*	*	*
Previously attended family support groups, *n* (%)	2 (4.30)	0 (0.00)	0.167	*	*	*	*	*	*
Relation to patient:									
Relation to patient, parent, *n* (%)	15 (32.60)	16 (37.21)	0.649	*	*	*	*	*	*
Relation to patient, child, *n* (%)	0 (0.00)	3 (7.00)	0.068	*	*	*	*	*	*
Relation to patient, sibling, *n* (%)	2 (4.35)	3 (6.98)	0.590	*	*	*	*	*	*
Relation to patient, spouse/partner, *n* (%)	28 (46.50)	20 (60.90)	0.174	*	*	*	*	*	*
Relation to patient, grandparent, *n* (%)	1 (2.30)	1 (2.20)	0.298	*	*	*	*	*	*
Well-being and functioning:									
WHO-5 well-being, total score, mean (s.d.)	63.57 (13.86)	64.93 (17.02)	0.680	68.33 (15.43)	68.86 (17.55)	0.891	65.78 (17.80)	73.83 (11.19)	0.062
WHO-5 well-being, item 1 “cheerful mood”, mean (s.d.)	3.46 (0.75)	3.62 (0.85)	0.345	3.53 (0.84)	3.76 (0.95)	0.282	3.48 (0.94)	3.83 (0.48)	0.104
WHO-5 well-being, item 2 “calm and relaxed”, mean (s.d.)	3.20 (0.86)	3.33 (1.05)	0.502	3.22 (0.96)	3.43 (1.07)	0.380	3.19 (1.04)	3.88 (0.54)	0.005
WHO-5 well-being, item 3 “active and vigorous”, mean (s.d.)	3.00 (0.87)	3.11 (0.99)	0.550	3.47 (0.94)	3.43 (0.80)	0.846	3.15 (1.03)	3.07 (1.24)	0.025
WHO-5 well-being, item 4 “rested on waking”, mean (s.d.)	2.98 (1.20)	2.86 (1.28)	0.648	3.28 (1.06)	3.24 (0.95)	0.884	3.07 (1.24)	3.42 (1.10)	0.304
WHO-5 well-being, item 5 “interest in daily life”, mean (s.d.)	3.28 (1.00)	3.29 (1.09)	0.989	3.61 (0.96)	3.54 (0.87)	0.743	3.44 (0.97)	3.63 (0.77)	0.470
FAD, problem solving, mean (s.d.)	1.85 (0.41)	1.77 (0.51)	0.590	1.76 (0.50)	1.92 (0.34)	0.235	1.70 (0.45)	1.66 (0.72)	0.868
FAD, communication, mean (s.d.)	2.49 (0.28)	2.44 (0.22)	0.473	2.49 (0.24)	2.51 (0.19)	0.782	2.44 (0.22)	2.56 (0.25)	0.094
FAD, affective responsiveness, mean, (s.d.)	2.62 (0.37)	2.62 (0.28)	0.995	2.63 (0.40)	2.73 (0.26)	0.352	2.73 (0.22)	2.75 (0.27)	0.791
FAD, affective involvement, mean (s.d.)	3.28 (0.43)	3.32 (0.51)	0.746	3.46 (0.51)	3.20 (0.60)	0.137	3.57 (0.30)	3.57 (0.69)	0.991
FAD, general functioning, mean (s.d.)	2.35 (0.13)	2.43 (0.15)	0.055	2.38 (0.17)	2.44 (1.18)	0.203	2.39 (0.19)	2.40 (0.20)	0.769
FAS, total score, mean (s.d.)	25.52 (13.66)	25.02 (11.82)	0.857	19.34 (15.03)	23.86 (12.44)	0.168	23.14 (19.50)	19.96 (9.66)	0.345
Psychiatric symptom burden:									
SCL-90, GSI, mean (s.d.)	0.55 (0.41)	0.53 (0.39)	0.861	*	*	*	*	*	*
SCL-90, somatization, mean (s.d)	0.54 (0.51)	0.54 (0.51)	0.992	*	*	*	*	*	*
SCL-90, anxiety, mean (s.d)	0.34 (0.37)	0.39 (0.41)	0.552	*	*	*	*	*	*
SCL-90, interpersonal sensitivity, mean (s.d)	0.57 (0.54)	0.56 (0.36)	0.968	*	*	*	*	*	*
SCL-90, phobic anxiety, mean (s.d)	0.15 (0.21)	0.16 (0.20)	0.799	*	*	*	*	*	*
SCL-90, obsessive cumpulsive, mean (s.d)	0.75 (0.66)	0.68 (0.53)	0.569	*	*	*	*	*	*
SCL-90, depression, mean (s.d)	0.85 (0.64)	0.75 (0.60)	0.492	*	*	*	*	*	*
SCL-90, anger hostility, mean (s.d)	0.42 (0.40)	0.34 (0.29)	0.325	*	*	*	*	*	*
SCL-90, paranoid ideation, mean (s.d)	0.34 (0.42)	0.28 (0.44)	0.482	*	*	*	*	*	*
SCL-90, psychoticism, mean (s.d)	0.28 (0.40)	0.23 (0.27)	0.492	*	*	*	*	*	*

Age, participant age; Sex, female gender; Education years, total years of formal education; Relation to patient, relationship to the patient (parent/child/sibling/spouse or partner/grandparent); Education level, highest level of education completed (non-tertiary or tertiary); History of psychiatric treatment, previous psychiatric care (yes); Employed or in education, currently employed or enrolled in education; Not in employment, unemployed or out of education; Previously attended family support groups, prior participation in psychoeducational or support programs for relatives; WHO-5 well-being, World Health Organization 5-item Well-being Index total score and individual items; FAD, Family Assessment Device; FAS, The Family Attitude Scale; SCL-90, Symptom Checklist-90 dimensions: GSI, Global Severity Index; *n*, number; s.d., standard deviation; %, percentage. * Variables on sociodemographic characteristics, relation to patient, and psychiatric symptom burden were collected at baseline only. No data or *p*-values are available for post-intervention and 9-month follow-up.

**Table 2:** Outcomes at postintervention and 9-month follow-up among relatives of patients with depression according to intervention group.

	Time: Baseline → Postintervention *p*#: *p*-Value for Time Alone *p*: *p*-Value for Time by Treatment Groups		Time: Postintervention → 9 Months Follow-Up *p*#: *p*-Value for Time Alone *p*: *p*-Value for Time by Treatment Groups		Time: Baseline → 9 Months Follow-Up *p*#: *p*-Value for Time Alone *p*: *p*-Value for Time by Treatment Groups		Time: Baseline → Postintervention → 9 Months Follow-Up *p*#: *p*-Value for Time Alone *p*: *p*-Value for Time by Treatment Groups	
Outcome	Model 1 (Unadjusted)	Model 2 (Adjusted)	Model 1 (Unadjusted)	Model 2 (Adjusted)	Model 1 (Unadjusted)	Model 2 (Adjusted)	Model 1 (Unadjusted)	Model 2 (Adjusted)
WHO well-being scale total score	*p#* = 0.074, F = 3.284 *p =* 0.736, F = 3.284	*p#* = 0.873, F = 0.026 *p =* 0.869, F = 0.027	*p#* = 0.675, F = 0.178 *p =* 0.675, F = 0.178	*p#* = 0.528, F = 0.406 *p =* 0.660, F = 0.196	*p#* = 0.316, F = 1.028 *p =* 0.532, F = 0.396	*p#* = 0.480, F = 0.508 *p =* 0.741, F = 0.111	*p#* = 0.390, F = 0.943 *p =* 0.868, F = 0.132	*p#* = 0.693, F = 0.355 *p =* 0.867, F = 0.133
WHO-5 item 1	*p#* = 0.334, F = 0.947 *p =* 0.890, F = 0.019	*p#* = 0.921, F = 0.010 *p =* 0.738, F = 0.113	*p#* = 0.471, F = 0.530 *p =* 0.179, F = 1.872	*p#* = 0.865, F = 0.029 *p =* 0.229, F = 1.491	*p#* = 0.951, F = 0.004 *p =* 0.297, F = 1.109	*p#* = 0.274, F = 1.225 *p =* 0.148, F = 2.168	*p#* = 0.548, F = 0.592 *p =* 0.178, F = 1.768	*p#* = 0.649, F = 0.417 *p =* 0.223, F = 1.532
WHO-5 item 2	*p#* = 0.467, F = 0.534 *p =* 1.000, F = 0.000	*p#* = 0.321, F = 0.998 *p =* 0.750, F = 0.102	*p#* = 0.615, F = 0.257 *p =* 0.983, F = 0.000	*p#* = 0.912, F = 0.012 *p =* 0.964, F = 0.002	*p#* = 0.698, F = 0.152 *p =* 0.206, F = 1.645	*p#* = 0.766, F = 0.089 *p =* 0.276, F = 1.216	*p#* = 0.813, F = 0.174 *p =* 0.765, F = 0.231	*p#* = 0.899, F = 0.080 *p =* 0.773, F = 0.218
WHO-5 item 3	***p#* = 0.007**, F = 7.742 *p =* 0.450, F = 0.576	*p#* = 0.721, F = 0.129 *p =* 0.521, F = 0.417	*p#* = 0.461, F = 0.554 *p =* 0.461, F = 0.554	*p#* = 0.629, F = 0.237 *p =* 0.517, F = 0.427	*p#* = 0.099, F = 2.835 *p =* 0.454, F = 0.570	*p#* = 0.781, F = 0.078 *p =* 0.421, F = 0.660	*p#* = 0.119, F = 2.201 *p =* 0.792, F = 0.221	*p#* = 0.823, F = 0.196 *p =* 0.808, F = 0.202
WHO-5 item 4	*p#* = 0.169, F = 1.929 *p =* 0.554, F = 0.354	*p#* = 0.387, F = 0.759 *p =* 0.585, F = 0.301	*p#* = 0.470, F = 0.531 *p =* 0.618, F = 0.253	*p#* = 0.246, F = 1.387 *p =* 0.486, F = 0.494	*p#* = 0.578, F = 0.313 *p =* 0.105, F = 2.725	*p#* = 0.508, F = 0.445 *p =* 0.295, F = 1.124	*p#* = 0.310, F = 1.186 *p =* 0.298, F = 1.227	*p#* = 0.477, F = 0.747 *p =* 0.334, F = 1.111
WHO-5 item 5	*p#* = 0.163, F = 1.987 *p =* 0.702, F = 0.148	*p#* = 0.543, F = 0.374 *p =* 0.798, F = 0.066	*p#* = 0.692, F = 0.159 *p =* 0.907, F = 0.014	*p#* = 0.877, F = 0.024 *p =* 0.759, F = 0.096	*p#* = 0.566, F = 0.333 *p =* 0.794, F = 0.069	*p#* = 0.751, F = 0.102 *p =* 0.967, F = 0.002	*p#* = 0.865, F = 0.130 *p =* 0.894, F = 0.099	*p#* = 0.742, F = 0.282 *p =* 0.810, F = 0.195
Family attitude scale	***p#* = 0.010**, F = 7.023 *p =* 0.147, F = 2.149	*p#* = 0.640, F = 0.221 *p =* 0.280, F = 1.186	*p#* = 0.328, F = 0.978 *p =* 0.292, F = 1.136	*p#* = 0.564, F = 0.338 *p =* 0.355, F = 0.875	*p#* = 0.328, F = 0.978 *p =* 0.292, F = 1.136	*p#* = 0.564, F = 0.338 *p =* 0.355, F = 0.875	*p#* = 0.175, F = 1.818 *p =* 0.163, F = 1.897	*p#* = 0.699, F = 0.307 *p =* 0.257, F = 1.379
FAD (family assessment device) problem-solving	*p#* = 0.970, F = 0.001 *p =* 0.129, F = 2.406	*p#* = 0.116, F = 2.593 *p =* 0.110, F = 2.689	*p#* = 0.703, F = 0.150 *p =* 0.501, F = 0.470	*p#* = 0.599, F = 0.287 *p =* 0.345, F = 0.940	*p#* = 0.325, F = 0.967 *p =* 0.704, F = 0.148	*p#* = 0.671, F = 0.186 *p =* 0.921, F = 0.010	*p#* = 0.613, F = 0.335 *p =* 0.504, F = 0.541	*p#* = 0.427, F = 0.707 *p =* 0.407, F = 0.769
FAD communication	*p#* = 0.531, F = 0.399 *p =* 0.630, F = 0.235	*p#* = 0.068, F = 3.543 *p =* 0.704, F = 0.147	*p#* = 0.527, F = 0.413 ***p =* 0.039**, F = 4.834	*p#* = 0.485, F = 0.508 *p =* 0.084, F = 3.337	*p#* = 0.942, F = 0.005 *p =* 0.096, F = 2.996	*p#* = 0.647, F = 0.215 *p =* 0.099, F = 2.980	*p#* = 0.862, F = 0.149 *p =* 0.115, F = 2.316	*p#* = 0.461, F = 0.783 *p =* 0.171, F = 1.872
FAD affective responsiveness	*p#* = 0.288, F = 1.162 *p =* 0.454, F = 0.573	*p#* = 0.216, F = 1.585 *p =* 0.644, F = 0.217	*p#* = 0.798, F = 0.067 *p =* 0.288, F = 1.186	*p#* = 0.861, F = 0.031 *p =* 0.360, F = 0.882	*p#* = 0.264, F = 1.305 *p =* 0.931, F = 0.008	*p#* = 0.624, F = 0.247 *p =* 0.991, F = 0.000	*p#* = 0.317, F = 1.183 *p =* 0.492, F = 0.688	*p#* = 0.891, F = 0.092 *p =* 0.517, F = 0.640
FAD affective involvement	*p#* = 0.508, F = 0.447 ***p =* 0.022,** F = 5.710	*p#* = 0.729, F = 0.122 ***p =* 0.041**, F = 4.516	*p#* = 0.585, F = 0.307 *p =* 0.258, F = 1.352	*p#* = 0.780, F = 0.080 *p =* 0.223, F = 1.595	*p#* = 0.229, F = 1.524 *p =* 0.890, F = 0.020	*p#* = 0.914, F = 0.012 *p =* 0.689, F = 0.165	*p#* = 0.277, F = 1.325 *p =* 0.271, F = 1.325	*p#* = 0.847, F = 0.166 *p =* 0.280, F = 1.294
FAD general functioning	*p#* = 0.439, F = 0.611 *p =* 0.531, F = 0.400	*p#* = 0.394, F = 0.744 *p =* 0.562, F = 0.343	*p#* = 0.600, F = 0.283 *p =* 0.289, F = 1.183	*p#* = 0.268, F = 1.308 *p =* 0.270, F = 1.294	*p#* = 0.987, F = 0.000 *p =* 0.364, F = 0.857	*p#* = 0.180, F = 1.919 *p =* 0.388, F = 0.778	*p#* = 0.698, F = 0.354 *p =* 0.251, F = 1.433	*p#* = 0.315, F = 1.196 *p =* 0.320, F = 1.178

Results are presented from repeated-measures analyses of covariance (ANCOVA). Model 1 is unadjusted, and Model 2 is adjusted for age and sex. *p*# indicates the effect of time across groups, and *p* the time × group interaction effect. Significant *p*-values are shown in bold. WHO-5, World Health Organization 5-item Well-Being Index; FAD, Family Assessment Device.

## Data Availability

The data presented in this study are available on reasonable request from the corresponding author. The data is not publicly available due to privacy restrictions.
